# The Promise and Challenge of Genetic Biocontrol Approaches for Malaria Elimination

**DOI:** 10.3390/tropicalmed8040201

**Published:** 2023-03-29

**Authors:** Stephanie James, Michael Santos

**Affiliations:** Foundation for the National Institutes of Health, North Bethesda, MD 20852, USA

**Keywords:** genetic biocontrol, malaria prevention, malaria elimination, transmission blocking, mosquito vectors, biological control, genetic modification

## Abstract

Malaria remains an ongoing public health challenge, with over 600,000 deaths in 2021, of which approximately 96% occurred in Africa. Despite concerted efforts, the goal of global malaria elimination has stalled in recent years. This has resulted in widespread calls for new control methods. Genetic biocontrol approaches, including those focused on gene-drive-modified mosquitoes (GDMMs), aim to prevent malaria transmission by either reducing the population size of malaria-transmitting mosquitoes or making the mosquitoes less competent to transmit the malaria parasite. The development of both strategies has advanced considerably in recent years, with successful field trials of several biocontrol methods employing live mosquito products and demonstration of the efficacy of GDMMs in insectary-based studies. Live mosquito biocontrol products aim to achieve area-wide control with characteristics that differ substantially from current insecticide-based vector control methods, resulting in some different considerations for approval and implementation. The successful field application of current biocontrol technologies against other pests provides evidence for the promise of these approaches and insights into the development pathway for new malaria control agents. The status of technical development as well as current thinking on the implementation requirements for genetic biocontrol approaches are reviewed, and remaining challenges for public health application in malaria prevention are discussed.

## 1. Introduction

Through global planning and intensive control efforts [1], the worldwide death toll of malaria was reduced by almost half between 2000 and 2015 [2,3]. Vector control measures, consisting primarily of insecticide-treated bed nets and indoor residual insecticide spraying, contributed by far the largest proportion of this reduction [2]. Since then, however, global progress has substantially declined, and malaria-related deaths have again begun to rise, particularly in the African region [4]. Malaria was reported to cause some 619,000 deaths worldwide in 2021, approximately 96% of which occurred in Africa [4]. As a result, the World Health Organization has issued warnings that progress toward important internationally agreed-upon malaria elimination milestones [4,5] is off course.

Declines in the effectiveness of current control tools due to increases in the insecticide resistance of mosquitoes and the drug resistance of parasites play a large role in this reversal [4]. Consequently, there have been multiple calls to improve methods for malaria treatment and prevention (e.g., [6,7,8]). Given the widely recognized importance of vector control as a vital component of malaria control and elimination strategies, there is increased interest in the development of innovative methods to prevent disease transmission by mosquito vectors [9]. Among the prospective new methods for mosquito control are various genetic biocontrol technologies [10]. 

## 2. Genetic Biocontrol

Classical biocontrol, or biological control, involves the use of living organisms to manage a pest problem (see Box 1). Traditionally, the problem has most often been caused by an invasive species, and the biocontrol agent has been a natural enemy identified in the native territory of the pest species and introduced into the new area where the unwanted species has become invasive [11]. The biocontrol agent could, for example, be a predator, competitor, or pathogen that acts to reduce the pest population in its new location.

Box 1 **Glossary.**

*Classical biocontrol (biological control)*—the control of a pest through the release of a natural enemy of that pest

*Genetic biocontrol*—a type of biocontrol that uses genetic variants or genetically engineered forms of the pest organisms themselves as the biocontrol agent

*Gene drive*—a process that promotes the inheritance of certain genes from generation to generation so that they increase in frequency within a population

*Population replacement (population modification)*—a form of biocontrol that reduces the ability of the target vector species to transmit a pathogen

*Population suppression (population reduction)*—a form of biocontrol that reduces the population size of the target vector species

*Self-limiting gene drive*—a system that imposes a temporal restriction on the inheritance of the modification

*Self-sustaining gene drive*—a system that promotes persistence of the modification at high frequency within the target vector population

*Localizing (confined) gene drive*—a system that imposes a spatial restriction on the spread of the modification through a target vector population

Genetic biocontrol uses genetic variants or genetically engineered forms of the pest organisms themselves as new biocontrol agents. Often, only male insects are released. This has been found to be more cost efficient and minimizes the possibility that females might cause damage in some situations; for example, male mosquitoes do not bite. The release of these modified agents and their subsequent mating with the target pest species in the wild is expected to result in some desired changes in the native pest population. In the case of mosquito vectors of malaria, the modifications may aim either to reduce the size of the vector population by inhibiting their reproduction or survival (an approach termed population suppression or reduction) or to modify the mosquitoes to make them less competent to transmit the malaria parasite by inhibiting parasite development in the vector or changing vector behavior (population replacement or modification). Thus, genetic biocontrol offers an expanded range of options, including either reducing the target mosquito population, similarly to classical biocontrol, or altering the target species to reduce its vectorial capacity while the population size remains relatively unchanged. A combination of these strategies, in which the number of vector mosquitoes is decreased, and any that remain have a decreased potential to transmit disease, is also a possibility. 

## 3. Genetic Biocontrol Approaches

Several types of genetic biocontrol are being explored for disease-transmitting mosquitoes (reviewed in [12]), ranging from those that have no lasting effect after release to those aiming to introduce a persistent change in the characteristics of the local vector population. Self-limiting systems are designed to temporally limit the transmission of the modification to progeny resulting from the mating of the biocontrol agent with wild-type mosquitoes of the same species. The most stringent form of self-limiting system imposes complete inhibition of the reproductive capacity of the biocontrol agent. In this case, no viable progeny result, so the modification is not passed on to another generation when the agent is released and mates in the wild and thus disappears rapidly from the population. A less stringent self-limiting system would allow the genetic modification to be passed to approximately 50% of the offspring resulting from the mating of wild mosquitoes with those carrying the modification, according to Mendelian inheritance. This continuing dilution, plus any additional disadvantage incurred by fitness effects resulting from the modification, will cause the modification to decline in frequency within each subsequent generation. Over time, the modification will effectively disappear in the population in the absence of repeated releases of the biocontrol agent [12].

Other genetic biocontrol approaches involve some form of gene drive. Gene drive has been defined as a phenomenon of enhanced inheritance in which the prevalence of a genetic element or alternative form of a gene increases in subsequent generations, even in the presence of some fitness cost [13]. Self-limiting gene drive systems aim to increase the frequency of the modification within the target population for a period of time, after which the frequency will decline [12]. While self-limiting drive systems are designed to impose a temporal restriction on the persistence of the modification, self-sustaining gene drives are intended to pass the modification on at increasing frequency through subsequent generations so that it is maintained at a high level within the target population. Some self-sustaining drives (termed low or no threshold drives) have the capability to spread widely within interbreeding mosquito populations from only a few low-level introductions [12]—a potentially attractive characteristic that could substantially simplify and reduce the cost of delivery but which has raised certain safety concerns (discussed below). Localizing, or confined, drive systems aim to impose some spatial restriction on the spread of the modification through the target population [12]. Localizing systems may be either self-limiting or self-sustaining. For example, self-limiting drives may effectively be localizing because they do not persist long enough to spread widely. Alternatively, some localizing systems (termed high threshold drives) only become established and self-sustaining when the modified mosquitoes reach a certain frequency within the overall local population of the targeted species. These are expected to have limited ability to spread widely beyond the release area due to a decreasing probability of maintaining the threshold frequency with increasing distance from the release site.

## 4. Precedents for Biocontrol of Malaria Vectors

There is a great deal of practical experience with a population suppression method known as the Sterile Insect Technique (SIT) through programs spearheaded by the Food and Agriculture Organization (FAO), Rome, Italy and the International Atomic Energy Association (IAEA), Vienna, Austria [14]. SIT involves ongoing inundative releases of sterile male insects and has been used extensively against agricultural pests such as screwworm and medfly. In this case, the genetic modification results from the exposure of the insects to ionizing radiation, which damages their DNA and thereby renders them sterile. When these sterile males, the biocontrol agents, are released into the wild, their mating with native female insects will be nonproductive. If the sterile males are released in sufficient numbers to out-compete fertile wild males for mating with wild females, the result is a substantial decline in viable progeny and, thus, a local reduction in the overall numbers of the target species. The SIT method is being adapted to Aedes mosquitoes, known to transmit dengue and other arboviral diseases [14,15,16]. This method requires the ongoing production and release of large quantities of sterile male insects to sustain the desired population suppression effect.

The biotechnology company Oxitec, Abingdon, UK [17] has developed a program that uses genetic engineering, rather than radiation, to modify DNA and thus create mosquitoes and other insects for SIT-like population suppression. The first generation of their technology, the OX513A strain of male *Aedes aegypti* (Linnaeus in Hasselquist, 1762) mosquitoes, carried a repressible dominant lethal gene that was inherited by the offspring of mating with wild females, leading to the death of almost all progeny during larval or pupal development. This version was able to effect the suppression of *A. aegypti* populations in field trials [18,19]. As with classical radiation-based SIT, this effect was transient, and the cessation of the releases allowed the local *A. aegypti* population to regenerate rapidly. More recently, Oxitec has developed a second-generation product, the OX5034 strain of *A. aegypti*, in which the repressible lethal gene causes lethality only to female progeny. With this strain, the modification is passed on according to Mendelian inheritance through viable male progeny. This version is considered superior in that it eliminates the need to sort out only male mosquitoes for release and should simplify scale-up production [20]. This second-generation product also has been shown to produce high levels of population suppression as a result of ongoing field releases [20] and has been approved for commercial use in Brazil [21]. This product is currently being field tested in Florida and has received additional regulatory approval from the US Environmental Protection Agency (EPA) for testing in California [22]. 

SIT-like biocontrol methods that do not utilize genetic engineering are also being investigated in *A. aegypti*. The Incompatible Insect Technique (IIT) is a population suppression method based on the ability of Wolbachia (Hertig, 1936) bacteria to induce cytoplasmic incompatibility [23], which prevents the formation of viable offspring. Cytoplasmic incompatibility is manifested when male mosquitoes infected with certain Wolbachia strains mate with a female that is uninfected or that is infected with a different Wolbachia strain, and results in the death of the resulting embryo. The release of only the Wolbachia-infected males into a wild mosquito population can therefore produce population suppression. An IIT product has been tested in California, where it demonstrated strong population suppression of *A. aegypti* [24], and this technology in *Aedes albopictus* (Skuse, 1895) is registered by the US EPA for commercial use as a pesticide in several US states [25]. Registration of a similar *A. aegypti* IIT product is currently under consideration [22]. Comparable Wolbachia-based population suppression products are being utilized elsewhere [26]. A method combining radiation-based SIT and Wolbachia-based IIT for population suppression also is under investigation [27,28]. Similar to classical SIT, these methods require the ongoing releases of high numbers of modified mosquitoes to sustain suppression. 

A different Wolbachia-based approach provides a relevant precedent for the efficacy of self-sustaining drive systems for population replacement. In this case, low numbers of Wolbachia-infected female *A. aegypti* are also released along with males. While uninfected females can only mate productively with uninfected males, the Wolbachia-infected females have the reproductive advantage of mating successfully with either Wolbachia-infected or uninfected males, passing the maternally transmitted bacteria on to their viable offspring and thus spreading it within the local mosquito population. The Wolbachia infection can be maintained indefinitely in the target mosquito population if the frequency remains above a certain threshold (an example of a high-threshold method). Because Wolbachia infection has been shown to inhibit the development of a variety of arboviruses within the mosquito vector [29,30,31], this forms the basis of a population replacement strategy to reduce disease transmission. This method has been shown to significantly reduce dengue transmission in a randomized cluster-controlled trial conducted in Indonesia [32] and is being applied in multiple countries [33,34]. 

## 5. Genetic Biocontrol Options for Malaria Vectors

Some of the methods described above are also being tested in Anopheles malaria vectors, but the results are far more preliminary. Studies of radiation-based SIT for control of *Anopheles arabiensis* (Patton, 1905) in a region along the banks of the Nile have been conducted [35]. Efforts have begun to adapt the Oxitec technology to *Anopheles stephensi* (Liston, 1901) [36], a common malaria vector in Asia and the Middle East that has recently invaded the Horn of Africa [37]. Based on results in Aedes mosquitoes, these methods are likely to be effective if successfully adapted to anophelines. However, as noted above, they will be subject to the requirement for frequent releases to maintain efficacy.

The use of Wolbachia or other symbionts to effect population replacement in Anopheles vectors is also being explored. Partial protection has been noted in the laboratory against Plasmodium species in Wolbachia-infected anophelines [38,39] and *A. aegypti* [40]. A Wolbachia strain found to occur naturally in *Anopheles gambiae* (Giles, 1902) mosquitoes in Mali has been shown to decrease *Plasmodium falciparum* (Welch, 1897) sporozoite development in membrane feeding studies and to correlate with reduced Plasmodium infection in field-collected mosquitoes [41]. Nevertheless, several challenges remain for the successful field application of a Wolbachia-based population replacement approach to malaria control, including the demonstration that Wolbachia can induce cytoplasmic incompatibility in *A. gambiae*, as would be necessary for the establishment and spread in the local vector population [38,42]. Other inherited microbial symbionts are also being explored for their ability to suppress Plasmodium transmission [43,44]. 

Gene-drive-modified mosquitoes (GDMMs) have been recognized as a potentially transformative new tool for the control and elimination of malaria and other mosquito-borne diseases [45], having the potential to deliver area-wide population-level control that is both cost-effective and durable. Most gene drive research is currently focused on mosquitoes of the *A. gambiae* species complex, which historically have been important vectors in Africa, where the malaria burden remains greatest [46,47]. The earliest reports of success were made possible by the use of a system that recapitulates the function of natural homing endonuclease genes found in a wide range of microbes as well as eukaryotic mitochondria and chloroplasts. Within the sperm or egg cells, a homing endonuclease gene located on one chromosome produces a nuclease enzyme that recognizes and cleaves specific sequences of DNA on the homologous chromosome and the cell’s own machinery then repairs the break by copying and inserting the endonuclease gene sequence into the site. This transforms an organism that would ordinarily be heterozygous for the nuclease gene into one that is homozygous. The nuclease gene will thus be inherited by all of the organism’s progeny. As this effect occurs in each subsequent generation, the nuclease gene rapidly spreads within the population [48]. Two decades ago, it was hypothesized that if homing endonuclease genes could be targeted to insert into and inactivate host genes essential for reproduction, this could result in population suppression [49]. It was subsequently shown in a model system that homing endonucleases occurring naturally in other organisms retained their function when transferred into *A. gambiae* mosquitoes [50]; however, reprogramming such natural homing endonucleases to recognize native target sequences in the mosquito genome proved too cumbersome for practical use in a genetic biocontrol approach. 

Several years later, it was proposed that natural homing endonuclease activity could be mimicked by a genetic engineering method in which a bacterial Cas9 endonuclease is led to cut a specific target DNA sequence by a complementary RNA guide, creating a homology-driven repair system that could be used either to inactivate essential genes or to carry new transgenes into the target genome [51]. Then, in 2015, the RNA-guided Cas9 system was demonstrated to work in Anopheles mosquitoes to generate homing gene drive systems for either: carrying cargo genes coding for single-chain antibodies that inhibit the development of *Plasmodium falciparum* parasites [52]; or inactivating female fertility genes [53]. These two early studies suggested the plausibility of creating self-sustaining gene drive systems for population replacement or population suppression, respectively, of malaria vectors. The two approaches have since been further expanded and refined. The *A. gambiae* gene doublesex, which is involved in sex determination, has been identified as an attractive target for both reducing mosquito numbers and altering their behavior. Female mosquitoes in which gene drive has disrupted the doublesex gene on both chromosomes show complete sterility as well as male-like morphologic changes that make them unable to bite and feed on blood [54]. Additionally, several potential effector mechanisms have been identified that could be used to inhibit parasite development in the mosquito vector, including antibodies that disable the parasite, antimicrobial peptides that kill it, or blockers of some critical mosquito–parasite interaction [55,56,57]. 

The research and development pathway for genetically modified mosquitoes specified by the World Health Organization [12] calls for trials initially conducted under physical confinement (containment), as in indoor insectaries or large cages. Reports of success are already coming from such trials. Following low-level introduction, an RNA-guided Cas9 construct targeting doublesex was found to spread rapidly within caged populations of *A. gambiae* mosquitoes and to cause complete suppression of the mosquito population in large indoor cages that simulate the field environment [54,58]. An RNA-guided Cas9 construct expressing anti-Plasmodium single-chain antibody fragments likewise has been found to spread rapidly in small cage populations of *A. stephensi* [59] and *A. gambiae* [60]. Modeling predicts that self-sustaining population suppression or population replacement systems could be effective for large-scale coverage at the national or multinational level [61,62,63].

Other methods to create self-sustaining gene drives are also under investigation, although these efforts are far more preliminary. These include drives that would cause population suppression by biasing the sex ratio of progeny toward all males. This type of drive has been shown experimentally to eliminate females in a population of *A. gambiae* [64]. Modeling supports the expectation that Anopheles mosquitoes modified with such male-biasing homing drives should reduce vector numbers as well as malaria transmission under a range of conditions [65,66]. Attempts to engineer drive systems that recapitulate toxin-antidote systems naturally occurring in other insects, which drive by inhibiting a maternally transmitted lethal trait, have been successful in Drosophila but less so in mosquitoes (e.g., [67,68,69]). 

Efforts to create gene drive systems that are temporally and/or spatially limited are likewise preliminary, and their utility in Anopheles mosquitoes remains largely theoretical at this time (for reviews, see [12,70,71,72]). However, confinable drive systems have been reported in *A. aegypti* [73,74]. 

## 6. Development Pathway

The WHO has provided comprehensive guidance for the testing of genetically modified mosquitoes, including GDMMs [12]. This calls for a phased pathway that begins under containment. Research will only extend beyond this phase if certain efficacy and safety characteristics are achieved. The conditions necessary for the containment of genetically and gene-drive-modified arthropods have been extensively detailed [75], and considerations for testing of GDMMs’ efficacy and safety at this early phase have been discussed [12,76,77]. According to the WHO guidance, the second phase of testing would be conducted in the field under conditions of physical and/or ecological confinement, and only if efficacy and safety requirements continue to be met would testing proceed to the third phase of open field testing. The guidance specifies a need for comprehensive risk assessment and appropriate stakeholder authorization before proceeding through each phase of testing and describes expectations for each of these activities as well as the applicable levels of regulatory oversight. The fourth phase of testing is composed of ongoing monitoring and surveillance for efficacy and safety conducted after the modified mosquitoes have been deployed in the context of a public health program. Other useful information can be found in the WHO guidance on the use of SIT against Aedes-borne diseases [16], which also discusses potential considerations for product licensure, deployment, and post-licensure evaluation.

## 7. Challenges for Implementation of Genetic Biocontrol

As mentioned above, population suppression approaches based on radiation, Wolbachia infection, or recombinant DNA-induced fertility reduction have already shown field efficacy in other insect species, including Aedes mosquitoes, and exemplary regulatory pathways exist. However, the transfer of these approaches to anophelines is still in the preliminary stages. Moreover, the application of these methods, which requires repeated releases of large numbers of modified mosquitoes for ongoing efficacy, is laborious and can be costly. As a result, to date, the demonstration of the utility of these methods has been geographically restricted to, for example, small cities or islands. Urban malaria is expected to be a growing global problem [78], and if these methods can be successfully adapted to malaria vectors, they may be able to make a meaningful contribution in that context. Improvements in all aspects of the supply chain, from manufacturing through delivery, will be needed if these methods are to be more widely useful, especially in rural and under-resourced areas that bear a heavy malaria burden. Research to address some of these needs is underway. For example, automation processes employing robotics for mass rearing and sex separation of Aedes mosquitoes are being tested, e.g., [24], although the potential applicability of these technically complex methods to a field setting remains unclear. Drone transport of mosquitoes has been piloted within fairly limited areas, e.g., [79], and may hold promise for extending delivery to otherwise difficult-to-reach areas. 

Wolbachia-based population replacement relies on the long-term establishment of the virus-inhibiting Wolbachia infection in the local mosquito population following releases of fewer modified mosquitoes over a more limited timeframe (usually a few months), which may offer production and delivery cost advantages. The regulatory pathway has been successfully navigated in several countries. However, it likewise has not yet been adapted to anophelines, and if it were, its utility would likely also be geographically localized (as to cities or islands) since it is expected that the threshold frequency necessary to maintain Wolbachia establishment would be difficult to sustain across large and rural areas.

Engineered gene drive approaches offer broad flexibility for the development of vector control tools. In theory, they can be used for both population suppression and population replacement, with a range of mechanisms available for each, and they can be designed to spread indefinitely or to be spatially or temporally limited. At this time, research on self-sustaining gene drives in anopheline malaria vectors is far more advanced than that on self-limiting or localizing approaches; however, their eventual utility in malaria control programs still faces technical as well as social hurdles. 

Gene drive technologies were originally envisioned as a “last-mile” mechanism to provide durable and low-cost interruption of disease transmission contributing to malaria elimination in regions such as Africa, a goal for which currently available control methods have thus far proven insufficient [4,80]. As research began to demonstrate that such self-sustaining gene drive technologies are feasible and research moved further toward field testing, public concerns arose about their technical limitations as well as their environmental and ethical implications [81]. 

One often-raised technical concern is the potential for the development of resistance mechanisms that will limit the timeframe over which gene drive approaches will be useful. Because reduced reproductive capacity is a substantial fitness cost, this concern is greater for those approaches aiming for population suppression than for population replacement [82]. Indeed, early cage studies with a gene drive targeting female fertility genes showed rapid induction of resistance due to mutations at the target site [83]. Researchers are examining several different and potentially complementary approaches to delay the appearance of resistance, including: aiming to disrupt multiple female fertility genes and/or multiple sequences within the same gene, utilizing promoters that bias toward homologous DNA repair and thus reduce the opportunity for mutation; and, aiming to disrupt genes that are highly conserved in the target mosquito species and/or cannot tolerate changes in their sequence without causing severe harm to the mosquito that would make a mutation less likely to be inherited (for review, see [72]). Methods are being developed to evaluate candidate gene drive targets for their resilience to resistance (e.g., [83,84,85]). For example, cage trials of a gene drive targeting doublesex described above found complete population suppression in *A. gambiae* mosquitoes with no evidence of resistance, presumably due to functional constraints in the target sequence. Nevertheless, it may be presumed that resistance to gene drives is likely to appear given sufficient time—as is also seen for insecticides used in mosquito control [86] as well as for antimalarial drugs [87]. With multiple potential targets and approaches available, it should be possible to manage resistance to GDMMs through the development of next-generation products, as is the strategy for insecticides and drugs. 

Another widely cited concern for self-sustaining gene drives is the presumed inability to stop their progress should an unanticipated adverse effect become apparent. For a small, ecologically confined release, as in the early phase of field testing, it may be possible to halt the drive by rigorous application of conventional insecticides [76]. It also has been suggested that the spread of self-sustaining population suppression gene drives could be limited by the purposeful introduction of resistance genes into the mosquito population, which would be strongly selected for at the expense of the drive [49]. Advances have been made in developing genetic methods to halt gene drives by replacing, modifying, or degrading them (e.g., [70,88,89]. However, none of these systems has been adequately tested in vivo, and more research is needed. Moreover, the public appetite to stop a gene drive that caused unintended consequences using another genetic approach must be considered. At this time, the best method to avoid the possibility of adverse effects is through prevention by comprehensive risk assessment prior to the release of gene-drive-modified mosquitoes. Considerations for risk assessment and risk management at each phase of testing, including identification of possible hazards for human and animal health or the environment, have been extensively described (e.g., [12,77,90,91], and currently are under consideration by the Convention on Biological Diversity [92]. In this regard, some have raised concerns as to whether existing regulatory and governance mechanisms are adequate to evaluate gene-drive-modified organisms (e.g., [93,94,95,96]), with some even suggesting that gene drive and other forms of gene editing research should motivate fundamental changes in scientific and regulatory processes to increase transparency and inclusivity in decision-making [95,97,98]. Others believe that current risk assessment and regulatory mechanisms will be adequate with some specific improvements (e.g., [90,99,100]). Pertinent regulatory frameworks and policies, and efforts to strengthen capacity for decision making, have been described elsewhere (e.g., [12,101,102]). 

Self-limiting and localizing drives may reduce some of the concerns that have been voiced about self-sustaining gene drive. It has been suggested that consideration be given to testing a self-limiting intermediate prior to a self-sustaining drive to gain experience and information that can inform risk assessment [12,76]. A number of different self-limiting and localizing drive options have been proposed, largely based on mathematical modeling, but have not yet progressed very far experimentally in anophelines. Assuming self-limiting and localizing approaches can be proven effective in malaria vectors, they must be submitted to the same regulatory oversight and rigorous risk assessment on a case-by-case basis that will be applied to self-sustaining gene-drive-modified mosquitoes. For example, it has been suggested that the spread of some self-limiting GDMM systems may be influenced by local conditions (e.g., [103]). Thus, some uncertainties are likely to confront new self-limiting, confined or self-sustaining GDMMs as they move toward large-scale implementation. Again, this understanding should be considered in the context of other malaria control tools, including insecticides, which carry some toxicity risks (e.g., [104]), and antimalarial drugs, which pose some risk of adverse effects [105]. 

How to appropriately communicate risk and take stakeholder opinion into account in the decision-making process about the implementation of gene drive products is a topic of intense debate, the extent of which is too great to fully describe here. Briefly, it is widely agreed that there are ethical considerations for the testing and use of all vector control measures. While many of these considerations and obligations are common to medical interventions more broadly, some distinct ethical questions focus on how the application of area-wide vector control methods affects individual autonomy since decisions about the implementation of vector control methods typically are made on a household or community-wide basis [106]. This necessitates a robust and inclusive plan for community engagement and authorization. The elements of such a process have been, and continue to be, studied (e.g., [12,76,107,108,109,110,111]). While it is considered standard practice to include input from those potentially affected by the intervention in the risk analysis and impact assessment processes, there is an ongoing discussion of how best to ensure that input is influential in decision making (e.g., [98,111,112,113]). Funders and developers of gene-drive-modified products have self-identified ethical principles to which they have pledged to adhere, which include engagement and benefit sharing [114,115,116]. 

Largely because of their potential to spread and persist within interbreeding populations, gene drive technologies have received particular attention with regard to how widely it is ethically appropriate to extend engagement activities. There are a variety of opinions about the role of various groups beyond the affected community in decision-making, including those that do not live at the intervention site but have some legitimate professional or personal interest in its conduct as well as those who lack any direct connection but express interest nonetheless (e.g., [12,81]). While it is largely agreed that ethical obligations devolve more toward interactive communication with these groups, the extent to which they should be included in processes for the governance of research involving gene editing and gene drive remains a subject of some debate [94,97,98,117,118,119]. Similar to risk and impact assessment, the response to these questions may best be approached on a case-by-case basis according to the characteristics of the specific product and its intended use.

## 8. Discussion

It is acknowledged that new tools are needed to achieve the global goals of malaria elimination. Genetic biocontrol approaches offer several important potential advantages over currently available malaria and vector control tools while also posing some different risks [12,81]. Among the advantages, they would be effective against outdoor and day-biting mosquitoes, as well as the indoor and night-biting mosquitoes targeted by indoor spraying and bed nets. The expectation that modified mosquitoes will exhibit the same natural behaviors as the targeted species would allow them to reach vector populations and larval breeding sites that have traditionally been the most difficult and expensive to access with insecticide-based interventions. They would provide protection that is not dependent upon human behavior, such as net usage, or on socioeconomic conditions influencing access to other malaria prevention or treatment options. They could both reduce malaria transmission in regions with active disease and protect against malaria re-establishment in regions where the disease has been eliminated. Additionally, they function via mating within the malaria vector population, which conveys a high level of target specificity that should make them environmentally friendly [12]. 

Beyond that, the different types of genetic biocontrol described here are expected to have different advantages and disadvantages for implementation. Their value proposition will depend on their potential public health contribution, their potential risks, and the activities and resources that will be required for their implementation. In general, non-driving and self-limiting products are expected to require more frequent releases than self-sustaining systems to maintain effectiveness, and localizing drive systems are expected to require more extensive releases than non-localizing drives to provide widespread coverage. These approaches will need to manufacture and deliver live mosquito products on an ongoing basis at the scale necessary to achieve and maintain epidemiological impact. Self-sustaining products are expected to have advantages related to ease of production and delivery as well as durability of effect, although these desirable characteristics also have been associated with concerns related to persistence in the environment.

All of the potential advantages of genetic biocontrol technologies for malaria elimination will remain theoretical until field trials can demonstrate entomological and epidemiological efficacy as well as safety to human health and the environment. This, of course, is the most proximal challenge for their inclusion in malaria control programs. As described above, several technical issues still remain to be addressed, but research is progressing rapidly in this regard. For genetically and gene-drive-modified biocontrol agents, clarification of the regulatory and stakeholder authorization requirements is also needed [12,101]. Substantial efforts are underway to develop suitable frameworks for risk assessment and stakeholder engagement, as well as to strengthen regulatory capacity in malaria-endemic countries to adequately evaluate emerging biocontrol technologies [12,91,101,102]. All forms of genetically modified mosquitoes will be subject to biosafety requirements under the Cartagena Protocol in most malaria-endemic countries (reviewed in [12,91,92,101]). While all new genetic biocontrol products are expected to undergo rigorous regulatory evaluation [12,101], it is likely that new self-sustaining gene drive products will be especially challenged to provide a scientifically sound and convincing demonstration of safety under conditions of uncertainty. It remains unknown at present whether self-limiting or localizing gene drive products will have any advantage for regulatory approval and public acceptance. 

Assuming safety and efficacy have been confirmed through phased testing, it is likely that the decision to incorporate any type of genetic biocontrol tool into a national control program also will be influenced by the perceived cost and differential advantage with respect to other malaria control tools. The sustainability of malaria control programs has been an enduring challenge. Maintaining adequate long-term funding for disease control efforts is notoriously difficult, and this has certainly been observed for malaria control [80,120]. Self-sustaining GDMM technologies were originally proposed as a possible solution to this long-standing problem. If self-sustaining gene drive products prove to provide low-cost, durable protection over several years as predicted, that characteristic should be attractive to national governments and other funders. For self-limiting technologies, ongoing funding will be required to maintain effectiveness. With SIT programs, for example, re-invasion can occur rapidly following any reduction or change in the pattern of releases, e.g., [121,122]. Depending on the number and extent of releases required to achieve the desired effect, however, self-limiting or confined drive systems may still provide some cost advantage in comparison to other vector control methods. 

The value proposition for genetic biocontrol technologies may well differ among countries, dependent upon local entomological and epidemiological conditions as well as the availability and utility of other malaria control tools. Currently, it is expected that new biocontrol technologies will complement, rather than replace, current methods in the global effort to eliminate malaria. Therefore, it will be important to consider how best to integrate biocontrol approaches with other malaria control measures. Different approaches might be more suitable to different transmission conditions, economic circumstances, and health goals. For example, non-driving and self-limiting or localizing approaches may prove applicable for malaria control over limited areas and a defined time frame. Characteristics of persistence and spread should increase the suitability of self-sustaining gene-drive-modified mosquitoes for use under more diverse circumstances, such as rural as well as urban environments, resource-challenged conditions, and situations where delivery of other tools has been difficult or disrupted. 

## 9. Conclusions

The development pathway for any new class of vector control product is complex; the WHO looks not only for proof of epidemiological efficacy but also at benefits and harms, resource implications, equity issues, and evidence of feasibility and acceptability [12,123,124]. Genetic biocontrol approaches for malaria are sufficiently novel that the mechanisms for addressing these requirements are not yet fully established. However, field successes of SIT, GM and Wolbachia-based biocontrol technologies in other mosquito species and results from cage studies of self-sustaining GDMMs in anopheline vectors provide a strong rationale to believe that genetic biocontrol approaches hold tremendous promise for malaria control and elimination (Table 1).

While research is moving rapidly, certain technical challenges remain to be addressed, and the basis for regulatory and stakeholder authorization must be clarified so that field trials can be appropriately conducted and evaluated. Although genetic biocontrol tools are still several years from practical application in malaria control and elimination programs, given the widely expressed need for innovative new approaches and favorable results thus far, they are well worth watching as important potential additions to the armamentarium of malaria interventions.

## Figures and Tables

**Table 1:** Biocontrol approaches with application to Aedes or Anopheles mosquito vectors.

Biocontrol Method	Current Status	References
Incompatible Insect Technique (IIT) for population suppression	Operational	[22,26]
Conditional lethal genetic modification for population suppression	Operational	[20,21]
*Wolbachia* bacteria for population replacement	Operational	[32,33,34]
Sterile Insect Technique(SIT)-IIT for population suppression	Large field tests	[28,125,126]
SIT for population suppression	Small field tests	[15,125]
Self-sustaining gene drive for population suppression	Indoor cage tests	[54,58]
Self-sustaining gene drive for population replacement	Indoor cage tests	[59,60]
Self-limiting or localizing gene drive for population suppression or replacement	Early research	[70,71,72,73,74]

## Data Availability

Not applicable.
